# Antiglycation and Antioxidant Properties of *Ficus deltoidea* Varieties

**DOI:** 10.1155/2020/6374632

**Published:** 2020-08-07

**Authors:** Nur Sumirah Mohd Dom, Nurshieren Yahaya, Zainah Adam, Nik Mohd Afizan Nik Abd. Rahman, Muhajir Hamid

**Affiliations:** ^1^Department of Microbiology, Faculty of Biotechnology and Biomolecular Sciences, Universiti Putra Malaysia, 43400 UPM Serdang, Seri Kembangan, Selangor, Malaysia; ^2^Department of Chemical Engineering Technology, Faculty of Engineering Technology, Universiti Malaysia Perlis, 02100 Sungai Chuchuh, Padang Besar, Perlis, Malaysia; ^3^Medical Technology Division, Malaysian Nuclear Agency, Bangi 43000, Kajang, Selangor, Malaysia; ^4^Department of Cell and Molecular Biology, Faculty of Biotechnology and Biomolecular Sciences, Universiti Putra Malaysia, 43400 UPM Serdang, Seri Kembangan, Selangor, Malaysia

## Abstract

The present study aimed to evaluate the potential of standardized methanolic extracts from seven *Ficus deltoidea* varieties in inhibiting the formation of AGEs, protein oxidation, and their antioxidant effects. The antiglycation activity was analyzed based on the inhibition of AGEs, fructosamine, and thiol groups level followed by the inhibition of protein carbonyl formation. The antioxidant activity (DPPH radical scavenging activity and reducing power assay) and total phenolic contents were evaluated. After 28 days of induction, all varieties of *Ficus deltoidea* extracts significantly restrained the formation of fluorescence AGEs by 4.55–5.14 fold. The extracts also reduced the fructosamine levels by 47.0–86.5%, increased the thiol group levels by 64.3–83.7%, and inhibited the formation of protein carbonyl by 1.36–1.76 fold. DPPH radical scavenging activity showed an IC_50_ value of 66.81–288.04 *μ*g/ml and reducing power activity depicted at 0.02–0.24 *μ*g/ml. The extent of phenolic compounds present in the extracts ranged from 70.90 to 299.78 mg·GAE/g. Apart from that, correlation studies between the activities were observed. This study revealed that seven varieties of *Ficus deltoidea* have the potential to inhibit AGEs formation and possess antioxidant activity that might be attributed to the presence of phenolic compounds.

## 1. Introduction

Diabetes mellitus is a common disease characterized by hyperglycemia that resulted in insulin deficiency or insulin resistance in the human body. Increased glucose levels in the blood lead to the development of advanced glycation end products (AGEs); these can result in many complications of diabetes. During protein glycation, amino groups of protein react with aldehydic or keto groups of reducing sugars to form Schiff bases before rearranging to form Amadori products. The products are stable compounds and undergo a series of irreversible reactions to form highly reactive substances such as glyoxal, methylglyoxal, dicarbonyl compounds, and 3-deoxyglucosone (3DG). Finally, these reactive carbonyls combine with the amino, sulfhydryl, and guanidine functional group of intracellular and extracellular proteins to form stable AGEs. This series of reactions produces irreversible AGEs products such as crosslinks, aromatic heterocycles, and oxidized compounds [1].

The accumulation of AGEs is toxic to cells. As it leads to increased oxidative stress, it promotes various diabetic complexities such as neuropathy, cataract, retinopathy, and atherosclerosis [2]. Protein glycation and AGEs formation can cause serious damage and be fatal to various organs by triggering dysfunction of the heart, eyes, kidneys, nerves, blood vessels, and impaired wound healing [3]. Several efforts have been explored to minimize the interactions of AGEs such as by inhibiting AGEs formation, blocking the actions of AGEs, or breaking preexisting AGE cross-links, controlling the glycemic index, modifying dietary intake, and using antioxidants and anti-AGE agents [4]. The discovery of new antiglycation agents is vital, and an inhibitor to protein glycation is currently in demand [5]. Medicinal plants possess the ability to reduce protein glycation and retard the modification of the biological activity of a protein that contributes to degradation and conversion to advanced glycation end products [3]. Inhibition of glycated end product formation is associated with the antioxidant potential and ability of the plant extracts [5].

A plant commonly used for medicinal purposes, the *Ficus deltoidea* Jack (Moraceae), is also known as *Mas Cotek, Serapat Angin*, and *Telinga Beruk* among locals in Malaysia. It is a diverse species of subgenus *Ficus*, section *Ficus*, and subsection *Frutescentiae* [6]. Not only that, there are more than 25 species reportedly available in the Sino-Himalayan and western Malesian region [7]. This plant is native to and widely distributed throughout Malaysia, Thailand, Sumatra, Java, Kalimantan, Sulawesi, and Maluku [8]. *Ficus deltoidea* has seven varieties, namely, var. *trengganuensis*, var. *kunstleri*, var. *intermedia*, var. *deltoidea*, var. *angustifolia*, var. *bilobata*, and var. *motleyana* which were found in the Malay Peninsula as described by Kochummen [9]. It is a shrub tree, easily recognized by the presence of golden dots on the upper lamina, has leafy twigs, and produces a milky juice. The var. *bilobata*, var. *angustifolia*, var. *kunstleri*, var. *intermedia*, var. *trengganuensis*, and var. *motleyana* have been reported to be commonly used in Malay traditional medicines [10].

The phytochemical constituents of *Ficus deltoidea* plant extracts were identified as phenolics, flavonoids, and tannins [11, 12]. Methanolic leaves and callus extracts of *Ficus deltoidea* var. *kunstleri* were reported to contain tannins, alkaloids, saponins, flavonoids, and phenolics [13]. Both vitexin and isovitexin were detected in the aqueous and methanolic extract of *Ficus deltoidea* leaves and thought to be responsible for the antidiabetic properties [14, 15]. A moretenol (C_30_H_50_O) isolate in methanolic *Ficus deltoidea* leaf extract has been detected using nuclear magnetic resonance and mass spectrometers [16]. A compound known as lupeol (C_30_H_50_O) exhibited antibacterial properties against *Staphylococcus aureus, Bacillus subtilis*, and *Escherichia coli* [17].

Traditionally, the decoctions of the leaves of *Ficus deltoidea* were taken by postnatal mothers to aid in the recovery of the uterus [18]. The plant has also been used in managing menstrual problems, as an antidiabetic treatment, to treat arthritis and also to boost the immune system. Besides, the leaves have been used to treat headaches, fevers [10], and toothaches [19, 20]. *Ficus deltoidea* has been associated with many pharmacological abilities such as antioxidant and antidiabetic properties [21, 22]. Adam et al. [23] have discovered that aqueous extract of *Ficus deltoidea* has an antihyperglycemic property which increases insulin secretion activity. Meanwhile, the methanolic and ethanolic extracts of *Ficus deltoidea* increased basal and insulin-mediated glucose uptake into adipocytes cells. The leaves of *Ficus deltoidea* possess anti-inflammatory properties [24] and are able to reduce nociception when tested on animal models [25]. The plant also serves as a wound-healing agent with enhanced fibroblast proliferation in rats [26] and has anticancer potential as it caused apoptosis in human ovarian carcinoma cell lines [27]. Apart from that, Zahra et al. [28] suggested the antiulcerogenic effect of *Ficus deltoidea* when tested on gastric walls of rats by inhibiting the submucosal edema and leucocytes infiltration of the submucosal layer. Findings by Amiera et al. [29] proposed the ability of the aqueous var. *deltoidea* and var. *angustifolia* extract as a uterotonic agent to induce the labor process and treat postpartum hemorrhage. Recent animal studies have shown that the leaves of *Ficus deltoidea* var. *trengganuensis*, var. *kunstleri*, var. *intermedia*, var. *deltoidea*, var. *motleyana*, and var. *bilobata* exhibited significant hypoglycemic effects in normal rats [30].

The discovery of a new antiglycation agent is vital in order to fight against protein glycation. The objectives of this study were to primarily investigate the inhibitory properties of seven varieties of methanolic extracts of *Ficus deltoidea* on AGEs and protein carbonyl formations. Fructosamine and protein thiol levels as well as its antioxidative effects were determined.

## 2. Materials and Methods

### 2.1. Plant Preparation

The methanolic crude extracts of *Ficus deltoidea* var. *trengganuensis* (FDT), *Ficus deltoidea* var. *kunstleri* (FDK), *Ficus deltoidea* var. *intermedia* (FDI), *Ficus deltoidea* var. *deltoidea* (FDD), *Ficus deltoidea* var. *angustifolia* (FDA), *Ficus deltoidea* var. *bilobata* (FDB), and *Ficus deltoidea* var. *motleyana* (FDM) were obtained from Atta-ur-Rahman Institute for Natural Product Discovery (AuRIns), UiTM Puncak Alam Campus (courtesy of Prof. Dr. Nor Hadiani Ismail). The fresh and cleaned leaves of *Ficus deltoidea* varieties were left to dry in the oven at 35°C for three days and ground into a powder. The powder was soaked in methanol (100 g/L), sonicated for 30 minutes, and filtered two times using a vacuum filter. The samples were frozen at −80°C and then lyophilized in a freeze dryer to remove the water residual. The crude extracts were kept in an amber glass bottle at −20°C before use.

All varieties of *Ficus deltoidea* were identified by Prof. Dr. Nashriyah Mat (expertise in agricultural plant science, physiology, and systematics (taxonomy) from Universiti Sultan Zainal Abidin (UniSZA)). The FDT, FDK, FDI, FDD, FDA, FDB, and FDB leave samples were collected from various places in Malaysia under voucher specimen numbers 00366, 00048, 00303, 00050, 00368, 00057, and 00313, respectively. The voucher specimens were deposited in the Herbarium of School of Agricultural Science and Biotechnology, Faculty of Bioresources and Food industry, UniSZA.

### 2.2. Antiglycation Assay

The protein glycation inhibitory activity was done according to the method by Sharma et al. [31] with minor modifications. A mixture of 500 *μ*L of bovine serum albumin (BSA) at 10 mg/mL and 400 *μ*L of 500 mM fructose in 100 mM phosphate-buffered saline (pH 7.4) was incubated in 0.02% (w/v) sodium azide at 37°C for 28 days followed by the addition of 100 *μ*L of plant extracts and quercetin (final concentration: 0.078–0.50 mg/mL). The reaction was terminated by adding 10 *μ*l of trichloroacetic acid (TCA), left at 4°C for 10 minutes followed by being centrifuged at 13,000 rpm for 5 minutes, and dissolved with alkaline phosphate buffer saline (pH 10). The formation of fluorescent AGEs was measured using a Multimode plate reader (Perkin Elmer, USA) at an excitation wavelength of 355 nm and an emission wavelength of 460 nm.

### 2.3. Protein Carbonyl Assay

The protein carbonyl content assay was conducted using a standard protocol by Levine and colleagues with minor modifications [32]. A total of 400 *μ*L of 2,4-dinitrophenylhydrazine (DNPH) (10 mM in 2.5 M HCl) was added with 100 *μ*L of glycated samples and incubated for 60 minutes in the dark. After incubation, 500 *μ*L of 20% (w/v) TCA was added to the solution for protein precipitation and left at 4°C for 5 minutes. After that, the solution was centrifuged at 10,000*g* for 10 minutes at 4°C, and the protein pellet was rinsed with a mixture of ethanol/ethyl acetate (1 : 1) three times and finally resuspended in 500 *μ*L of 6 M guanidine hydrochloride. The absorbance was captured at 370 nm using Multimode Plate Reader (Perkin Elmer, USA). The results were expressed as a percentage of inhibition (%).

### 2.4. Fructosamine Assay

The fructosamine level was determined using a nitroblue tetrazolium (NBT) assay by Ardestani and Yazdanparast [33]. An amount of 10 *μ*L of glycated samples was incubated with 90 *μ*L of 0.5 mM NBT in 100 mM carbonate buffer (pH 10.4) at 37°C. The absorbance was recorded at 530 nm using a Multimode Plate Reader (Perkin Elmer, USA). The level of fructosamine was determined using a different absorption time point at 10 and 15 minutes of 1-deoxy-1-morpholino-fructose (1-DMF) standard curve at a concentration of 0.31–5.0 mM.

### 2.5. Protein Thiol Assay

The protein thiol content in fructose-BSA glycated mixtures was assessed by Ellman's assay with slight modifications [34]. Ten microliters of glycated samples was incubated with 90 *μ*L of 5 mM 5,5′-dithiobis(2-nitrobenzoic acid) (DTNB) in 100 mM phosphate buffer (pH 7.4) at 25°C for 15 minutes. The absorbance of samples was measured at 412 nm using a Multimode Plate Reader (Perkin Elmer, USA). The concentration of free thiol was measured from the L-cysteine standard curve (0.031–5.0 mM). The amount of protein thiol was expressed as nmol L-cysteine/mg protein.

### 2.6. Antioxidant Assays

#### 2.6.1. 1,1-Diphenyl-2-Picryl-Hydrazyl (DPPH) Radical Scavenging Assay

Radical scavenging activity of the extracts against stable 1,1-diphenyl-2-picryl-hydrazyl (DPPH) was determined using the method by Shimada et al. [35] with some modifications. Briefly, 100 *μ*L of crude extracts (1 mg/mL) was dissolved in a solution of DMSO : ethanol (5% :95%). Then, the samples were added with 100 *μ*L of DPPH (0.2 mM) dissolved in ethanol and incubated in the dark at room temperature for 15 minutes. The absorbance of the mixture was determined at 517 nm using a Multimode Plate Reader (Perkin Elmer, USA). Ascorbic acid and quercetin were used as a positive control. The ability of the samples to scavenge DPPH radicals was determined as IC_50_ value.

#### 2.6.2. Reducing Power Assay

The reducing power assay was determined according to the Oyaizu method with some modifications [36]. A total of 100 *μ*L of samples (1.0 mg/mL) was dissolved in DMSO : H_2_O (5% : 95%). 250 *μ*L of 0.2 M phosphate buffer (pH 6) and 250 *μ*L 1% (w/v) of potassium ferricyanide were mixed into the vials and incubated at 50°C for 20 minutes. After incubation, 250 *μ*L of 10% TCA was added to the sample solution and centrifuged at 3,000 rpm for 10 minutes. Then, 62.5 *μ*L of supernatant was mixed with 62.5 *μ*L deionized water and 12.5 *μ*L of 0.1% (w/v) ferric chloride. The absorbance was read at 700 nm using a Multimode Plate Reader (Perkin Elmer, USA). Ascorbic acid and quercetin were used as a positive control in the experiment.

### 2.7. Total Phenolic Content Assay

The determination of phenolic content in the extracts was discovered using the Folin–Ciocalteu method [37] with minor alterations. Firstly, 0.2 mL of crude extract (1 mg/mL) was added with 2.8 mL distilled water and mixed thoroughly with 0.5 mL of Folin–Ciocalteu reagent for 3 minutes. Next, 2.0 mL of 20% (w/v) sodium carbonate was added to the mixtures and left for one-hour incubation in the dark at room temperature. The absorbance was figured at 650 nm using a Multimode Plate Reader (Perkin Elmer, USA). The total phenolic contents were figured from the gallic acid standard curve and expressed as mg of gallic acid equivalent per gram (mg GAE/g).

## 3. Results and Discussion

### 3.1. Effects of *Ficus deltoidea* Varieties on Antiglycation

**Figure 1:**
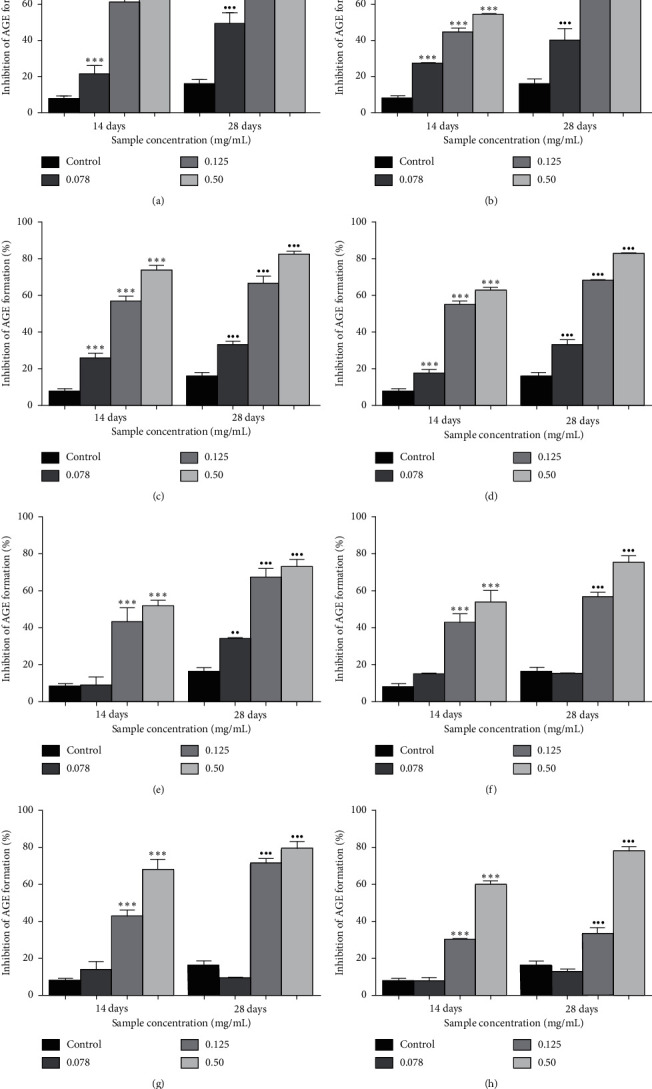
The effects of quercetin (a) and the extracts of *Ficus deltoidea* varieties, (b) *Ficus deltoidea* var. *trengganuensis*, (c) *Ficus deltoidea* var. *kunstleri*, (d) *Ficus deltoidea* var. *intermedia*, (e) *Ficus deltoidea* var. *deltoidea*, (f) *Ficus deltoidea* var. *angustifolia*, (g) *Ficus deltoidea* var. *bilobata,* and (h) *Ficus deltoidea* var. *motleyana* on the inhibition of AGEs formation (%). ^*∗*^*p* < 0.05,^*∗∗*^*p* < 0.01,^*∗∗∗*^*p* < 0.001,^*·*^*p* < 0.05,^*··*^*p* < 0.01,  and ^*···*^*p* < 0.001 when compared to the control at days 14 and 28 of study, respectively.

Figure 1 shows the effects of *Ficus deltoidea* varieties and quercetin on inhibition of AGEs formation. The inhibition of AGEs formation in fructose-glycated BSA was observed at 14 and 28 days and showed a significant increase in percentage inhibition of AGEs formation. Each value corresponds to the mean ± SD (*n* = 6). The results showed that *Ficus deltoidea* varieties and quercetin (0.078–0.50 mg/mL) significantly reduced the formation of AGEs in a concentration-dependent manner. A significant inhibition of the formation of AGEs was observed highest in quercetin at a concentration of 0.078, 0.125, and 0.50 mg/mL which evoked 2.85- (*p* < 0.001), 8.13- (*p* < 0.001), and 12.27- (*p* < 0.001) fold of inhibition, respectively, compared to the control at 14 days. At 28 days, the same trend was observed with 3.09-, 4.81-, and 5.78-fold of inhibition (*p* < 0.001).

The same trend of inhibition of AGEs formation was demonstrated in *Ficus deltoidea* varieties where at a concentration of 0.50 mg/mL, FDK showed the highest fold of inhibition at 9.66 (*p* < 0.001) followed by FDB (9.00, *p* < 0.001), FDI (8.24, *p* < 0.001), FDM (7.91, *p* < 0.001), FDT (7.09, *p* < 0.001), FDA (7.08, *p* < 0.001), and FDD; 6.73 (*p* < 0.001), respectively, compared to the control at 14 days of incubation. Meanwhile, at 28 days of incubation, the highest inhibition of AGEs formation was found in FDI (5.14), FDK (5.09), FDB (4.93), FDM (4.88), FDA (4.68), FDT (4.55), and FDD (4.55) by the fold of inhibition, respectively, compared with the control. All varieties showed significant inhibition of AGEs formation with *p* < 0.001.

### 3.2. Effects of *Ficus deltoidea* Varieties on Protein Oxidation

**Figure 2:**
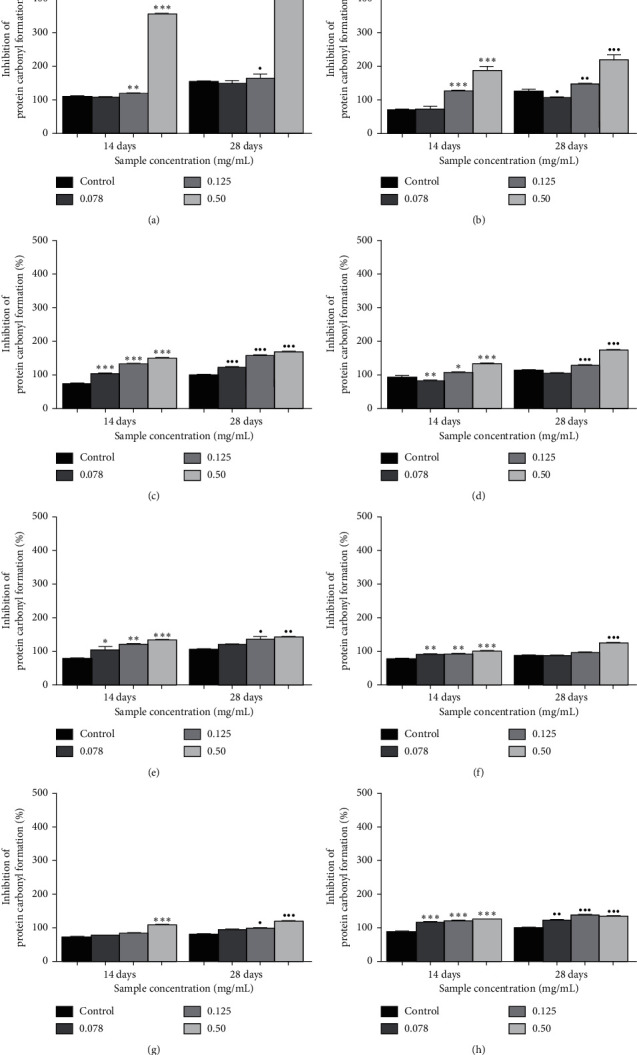
The effects of quercetin and (a) the extracts of *Ficus deltoidea* varieties, (b) *Ficus deltoidea* var. *trengganuensis*, (c) *Ficus deltoidea* var. *kunstleri*, (d) *Ficus deltoidea* var. *intermedia*, (e) *Ficus deltoidea* var. *deltoidea*, (f) *Ficus deltoidea* var. *angustifolia*, (g) *Ficus deltoidea* var. *bilobata,* and (h) *Ficus deltoidea* var. *motleyana* on the inhibition of protein carbonyl formation (%). Each value corresponds to the mean ± SD (*n* = 6). ^*∗*^*p* < 0.05,^*∗∗*^*p* < 0.01,^*∗∗∗*^*p* < 0.001,^*·*^*p* < 0.05,^*··*^*p* < 0.01,  and ^*···*^*p* < 0.001 when compared to the control at days 14 and 28 of study, respectively.

The effects of *Ficus deltoidea* varieties on glycation-induced protein oxidation were done by measuring the inhibition of protein carbonyl formation and thiol groups.  Figure 2 shows that the inhibition of protein carbonyl formation was significantly increased after 28 days of incubation. Quercetin is a positive control and showed a 3.04-fold of inhibition (*p* < 0.001) at a concentration of 0.50 mg/mL. FDT was found to possess the highest fold of inhibition at 1.76 (*p* < 0.001) followed by FDK (1.74; *p* < 0.001), FDI (1.53; *p* < 0.001), FDB (1.48; *p* < 0.001), FDA (1.41; *p* < 0.001), FDD (1.38; *p* < 0.001), and FDM (1.36; *p* < 0.001), respectively, when compared with the control at 28 days of incubation.

**Figure 3:**
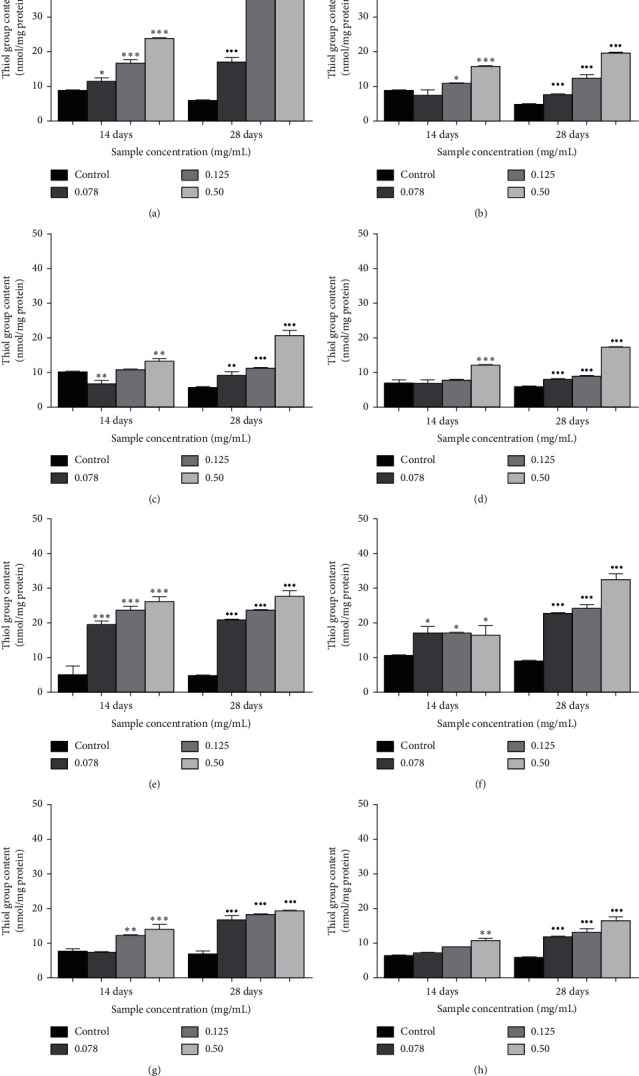
The effects of quercetin (a) and the extracts of *Ficus deltoidea* varieties, (b) *Ficus deltoidea* var. *trengganuensis*, (c) *Ficus deltoidea* var. *kunstleri*, (d) *Ficus deltoidea* var. *intermedia*, (e) *Ficus deltoidea* var. *deltoidea*, (f) *Ficus deltoidea* var. *angustifolia*, (g) *Ficus deltoidea* var. *bilobata,* and (h) *Ficus deltoidea* var. *motleyana* on the thiol group content (nmol/mg protein). Each value corresponds to the mean ± SD (*n* = 6). ^*∗*^*p* < 0.05,^*∗∗*^*p* < 0.01,^*∗∗∗*^*p* < 0.001,^*·*^*p* < 0.05,^*··*^*p* < 0.01,  and ^*···*^*p* < 0.001 when compared to the control at days 14 and 28 of study, respectively.

The effects of *Ficus deltoidea* varieties on glycation-induced oxidation of protein thiol are shown in Figure 3. After 28 days of incubation, the levels of thiol group were depleted in fructose-glycated BSA. However, when incubated with *Ficus deltoidea* varieties and quercetin (0.078–0.50 mg/ml), there was a significant increment of the thiol group levels in the system. The results showed that after 28 days of incubation, the highest percentage of inhibition of thiol group was found significantly in quercetin (86.1%; *p* < 0.001) followed by FDD (83.7%; *p* < 0.001), FDT (76.9%; *p* < 0.001), FDI (74.3%; *p* < 0.001), FDA (73.0%; *p* < 0.001), FDK (67.0%; *p* < 0.001), FDM (65.1%; *p* < 0.001), and FDB (64.3%; *p* < 0.001), respectively, at 0.50 mg/mL concentration when compared to the control.

**Figure 4:**
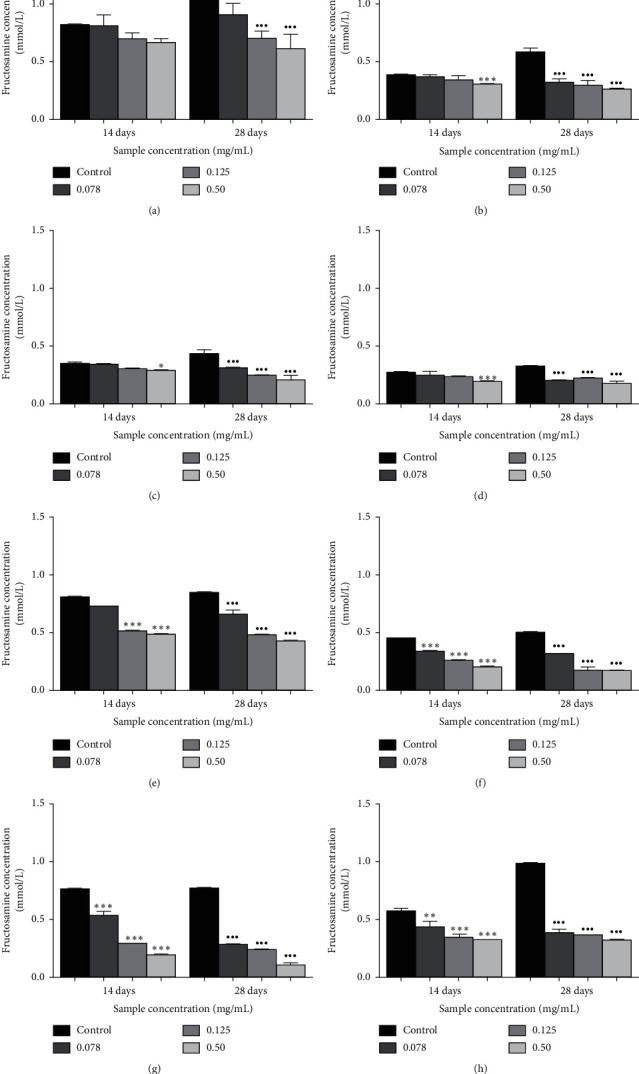
The effects of quercetin (a) and the extracts of *Ficus deltoidea* varieties, (b) *Ficus deltoidea* var. *trengganuensis*, (c) *Ficus deltoidea* var. *kunstleri*, (d) *Ficus deltoidea* var. *intermedia*, (e) *Ficus deltoidea* var. *deltoidea*, (f) *Ficus deltoidea* var. *angustifolia*, (g) *Ficus deltoidea* var. *bilobata,* and (h) *Ficus deltoidea* var. *motleyana* on the level of fructosamine (mmol/L). Each value corresponds to the mean ± SD (*n* = 6). ^*∗*^*p* < 0.05,^*∗∗*^*p* < 0.01,^*∗∗∗*^*p* < 0.001,^*·*^*p* < 0.05,^*··*^*p* < 0.01,  and ^*···*^*p* < 0.001 when compared to the control at days 14 and 28 of study, respectively.

The effects of *Ficus deltoidea* varieties on fructosamine levels are shown in Figure 4. The level of fructosamine in the BSA-fructose system was increased after 28 days of the incubation period. At day 28, the treatment of glycated samples with *Ficus deltoidea* extracts showed a significant reduction of fructosamine level with FDB at 86.5% (*p* < 0.001) followed by FDM (67.3%; *p* < 0.001), FDA (66.3%; *p* < 0.001), FDT (54.8%; *p* < 0.001), FDK (52.7%; *p* < 0.001), FDD (48.8%), and FDK (47.0%; *p* < 0.001), respectively, at 0.50 mg/mL. Meanwhile, quercetin decreased the level of fructosamine by 42.6% (*p* < 0.001).

### 3.3. DPPH Radical Scavenging Activity

DPPH scavenging activity of *Ficus deltoidea* varieties is shown in Table 1. Among the varieties, FDB depicted the highest scavenging activity (IC_50_: 66.81 ± 4.32 *μ*g/ml) followed by FDK (IC_50_: 76.80 ± 4.64 *μ*g/ml), FDI (IC_50_: 78.96 ± 6.25 *μ*g/ml), FDT (IC_50_: 103.95 ± 9.44 *μ*g/ml), FDD (IC_50_: 179.68 ± 6.81 *μ*g/ml), FDA (IC_50_: 203.92 ± 0.00 *μ*g/ml), and FDM (IC_50_: 288.04 ± 11.43 *μ*g/ml). Ascorbic acid (IC_50_: 1.3 ± 0.74 *μ*g/ml) and quercetin (IC_50_: 4.98 ± 1.58 *μ*g/ml) were used as a standard reference in this study.

### 3.4. Reducing Power Activity

Table 1 shows the reducing power activity of *Ficus deltoidea* varieties. The highest reducing power activity was found in FDI (0.24 ± 0.24 mg AAE/g) followed by FDK (0.21 ± 0.02 mg AAE/g), FDB (0.19 ± 0.06 mg AAE/g), FDT (0.10 ± 0.02 mg AAE/g), FDD (0.04 ± 0.02 mg AAE/g), FDM (0.03 ± 0.02 mg AAE/g), and FDA (0.02 ± 0.01 mg AAE/g). The standard curve of ascorbic acid was plotted to measure the reducing activity and quercetin was used as a positive control.

### 3.5. Total Phenolic Contents

Table 1 shows the total phenolic content in *Ficus deltoidea* varieties. According to Table 1, the highest total phenolic content in *Ficus deltoidea* extracts was found in FDB (299.78 ± 5.85 mg GAE/g) followed by FDI (190.30 ± 3.08 mg GAE/g), FDK (180.47 ± 3.25 mg GAE/g), FDT (163.28 ± 7.83 mg GAE/g), FDD (103.84 ± 5.93 mg GAE/g), FDM (78.44 ± 2.88 mg GAE/g), and FDA (70.90 ± 3.32 mg GAE/g). Quercetin was used as a standard reference and exhibited 1297.94 ± 17.66 mg GAE/g. The amount of phenolic contents was measured according to the standard curve of gallic acid (mg GAE/g).

### 3.6. Correlation Study

Table 2 shows Pearson's correlation analysis between (i) TPC and DPPH, (ii) TPC and reducing power activity, and (iii) TPC and inhibition of AGEs formation.

## 4. Discussion

The present research demonstrates the effects of *Ficus deltoidea* methanolic extracts from seven varieties on various parameters involved in glycation and protein oxidation. Glycation is a process of nonenzymatic reaction between reducing sugars (fructose or glucose) and amino groups of protein to form a Schiff base complex. The Schiff base formation is not stable and rearranged to produce irreversible Amadori products before being involved in further reactions to produce highly reactive carbonyl compounds. The dicarbonyls intermediates can react with amino, sulfhydryl, and guanidine functional groups resulting in browning, denaturation, and cross-linking of the targeted proteins [38, 39]. Besides, the dicarbonyl compounds can combine with lysine and arginine functional groups to form nonfluorescent advanced glycation end products such as *N*-carboxymethyllysine (CML) [40]. AGEs are complex materials that can be classified as fluorescent (pentosidine, crosslines, and imidazolones) and nonfluorescent (pyrraline, *N*-carboxymethyllysine, and *N*-carboxyethyllysine) cross-linking [41].

Quercetin is a flavonoid plant pigment, a potent hydroxyl radical, and superoxide anion scavenger. It is an established quencher for reactive oxygen species generated by any physical or chemical action that suppressed the rise in fluorescence of AGE formation [42]. During the inhibition of AGE formation, quercetin showed stronger inhibitory effects compared to aminoguanidine [43]. Quercetin was reported to inhibit the initial state of glycation by the low levels of HbA1c found in quercetin-treated hemoglobin samples and inhibited the post-Amadori glycation in human serum albumin-glucose and human serum albumin-methylglyoxal assays [42].

In this study, the inhibition of AGE formation was demonstrated to be the highest in FDI with 82.30% followed by FDK (81.50%), FDB (78.92%), FDM (78.15%), FDA (74.99%), FDT (72.93%), and FDD (72.83%) at the concentration of 5.0 mg/mL after 28 days of incubation. The results showed the inhibition of AGEs formation with FDI (5.14), FDK (5.09), FDB (4.93), FDM (4.88), FDA (4.68), FDT (4.55), and FDD (4.55) by fold, respectively, compared with the control. All varieties of *Ficus deltoidea* extracts inhibited significantly the AGE formation with *p* < 0.001. Inhibition of AGE formation in quercetin (5.0 mg/mL) was found significantly at 92.59% and 92.63% at 14 and 28 days which evoked 12.27- (*p* < 0.001) and 5.78- (*p* < 0.001) fold of inhibition, respectively, compared to the control of the same day. The results clearly showed that *Ficus deltoidea* extracts (0.078–0.125 mg/mL) significantly reduced the formation of AGEs in the BSA-fructose system throughout the study.

The amount of fructosamine was investigated by calculating the reducing activity of serum protein in the alkaline solution using the colorimetric test [44]. It was reported by Chompoo et al. [45] that the suppression of fructosamine and alpha-dicarbonyls levels could reduce the formation of AGEs. In this study, it was found that the highest percentage inhibition of fructosamine level was in FDB (86.5%) followed by FDM (67.3%), FDA (66.3%), FDT (54.8%), FDK (52.7%), FDD (48.8%), and FDI (46.9%), respectively, at 0.5 mg/mL concentration whereas quercetin showed a reduction of fructosamine at 42.6% after 28 days of incubation. During the initial state of glycation, the unstable Schiff complexes have formed, rearranged into Amadori products (fructosamine), and used as an indicator for controlling blood sugar levels in diabetes patients [40]. Fructose was found to be a faster reducing agent compared to glucose in suppressing the production of protein-bound fluorescence and various Amadori and cross-linking products at certain concentrations and ambient [46, 47].

Other than significantly increasing the levels of fructosamine when added to the fructose-BSA system, the *Ficus deltoidea* extracts also suppressed the protein carbonyl content and elevated the levels of protein thiol. In this study, the inhibition of protein carbonyl formation in the seven *Ficus deltoidea* varieties was observed. After 28 days of incubation, the carbonyl content of glycated BSA-fructose had increased. However, when treated with *Ficus deltoidea* extracts, the protein carbonyl content was significantly suppressed. The inhibition of protein carbonyl content was found highest in FDT (1.76; *p* < 0.001) followed by FDK (1.74; *p* < 0.001), FDI (1.53; *p* < 0.001), FDB (1.48; *p* < 0.001), FDA (1.41; *p* < 0.001), FDD (1.38; *p* < 0.001), and FDM (1.36; *p* < 0.001) by fold, respectively, when compared with the control at 28 days of incubation. Quercetin was used as a positive control to show 3.04-fold of inhibition (*p* < 0.001) at a 5.0 mg/mL concentration.

The level of protein thiol groups has subsided after 28 days of fructose and BSA incubation. However, a substantial increase in the level of thiol groups was recorded when *Ficus deltoidea* extracts and quercetin (0.078–0.50 mg/mL) were added to the glycated BSA fructose. The percentage of the prevention of thiol groups was found highest in quercetin (86.1%) followed by FDD (83.7%), FDT (76.9%), FDI (74.3%), FDA (73.0%), FDK (67.0%), FDM (65.1%), and FDB (64.3%), respectively, at 0.50 mg/mL concentration when compared to the control. After 28 days of incubation, the results showed that the seven varieties of *Ficus deltoidea* extracts significantly inhibited the reduction of protein thiol groups. During the glycation and glycoxidation processes, a reactive oxygen species was produced and oxidized with amino acid residues to form carbonyl derivatives and reduced an oxidative defense of protein by depletion of thiol groups, thus leading to the damage of the cellular proteins [48]. The production of superoxide anion (1,2- and 2,3-enolization of Schiff's base and oxidation of the enolate anion) is generated from the initial state of the glycation process [49, 50]. The mechanisms underlie antiglycation activity that has been suggested to break the inter- and intramolecular cross-linkages in the AGEs formation, inhibit the development of carbonyl or dicarbonyl groups in reducing sugars, Schiff bases, or Amadori compounds, and finally prevent the oxidative and nonoxidative cleavage of late-stage Amadori products [51].

The glycation of fructose sugar with BSA promotes protein oxidation and changes the structure of BSA associated with alterations of its biological characteristics [52]. Under hyperglycemic conditions, fructose was produced from sorbitol through glucose by the polyol pathway [53]. Fructose acts as an important precursor in the intracellular production of AGEs and engages in glycation better compared to glucose [54]. Fructose concentration was found to be higher in tissues of diabetic patients with active polyol pathway such as ocular lens, kidney, and peripheral nerves. Meanwhile, the thiol group of cysteine residues is receptive to oxidative attack by free radical damage to proteins, and the construction of disulfide bonds in protein aggregation resulted in the damage of enzymatic [55]. Not only that, the oxidation of amino acids such as lysine (Lys), arginine (Arg), and threonine (Thr) or secondary reaction of amino acid residues like cysteine (Cys) and histidine (His) with reactive carbonyl substances leads to the development of protein carbonyl derivatives [56].

It was proposed that the inhibition of glycated end product formation is associated with the ability of the antioxidant properties of the plant extracts to scavenge free radicals that were formed during the Maillard reaction in the glycation process [5]. Medicinal plant extracts possess the ability to reduce protein glycation and retard the modification of the biological activity of a protein that contributes to degradation as well as conversion to advanced glycation end products [3]. Furthermore, the protein glycation and oxidation process may interact synergistically in the progress of diabetic complications [57]. The traditional usage of herbal medicines has been shown to exhibit the effects of *in vitro* antioxidant, anti-inflammatory, and antiglycation properties [55]. Thus, inhibiting glycation should result in a beneficial effect on treating diabetes and at the same time fight against oxidative stress. Previous studies have revealed that both *Coccinia grandis* [58] and *Mesona chinensis* Benth extracts [52] inhibit AGEs formation and protein oxidation against fructose-induced protein glycation.

The free radical scavenging activity is reflective of the donation ability of hydrogen atoms or electrons in biological structures and configuration of the antioxidant compounds of the extracts. DPPH is a stable free radical compound and has been extensively applied to study the ability of free radical scavenging in natural products [59] which converts the purple color of DPPH radical to yellow DPPH-H at 517 nm. In other words, DPPH free radical scavenging activity refers to the amount of antioxidants needed to inhibit the DPPH radical concentration by half in 15 minutes (IC_50_). The lowest IC_50_ values indicated the highest antioxidant capacity of the extract. As DPPH radical is a stable compound, it is not affected by any side reactions, for instance, enzyme inhibition and metal chelation activity [60]. Among the *Ficus deltoidea* extracts, FDB showed the highest scavenging activity (IC_50_: 66.81 ± 4.32 *μ*g/ml) followed by FDK (IC_50_: 76.80 ± 4.64 *μ*g/ml), FDI (IC_50_: 78.96 ± 6.25 *μ*g/ml), FDT (IC_50_: 103.95 ± 9.44 *μ*g/ml), FDD (IC_50_: 179.68 ± 6.81 *μ*g/ml), FDA (IC_50_: 203.92 ± 0.00 *μ*g/ml), and FDM (IC_50_: 288.04 ± 11.43 *μ*g/ml). Ascorbic acid (IC_50_: 1.3 ± 0.74 *μ*g/ml) and quercetin (IC_50_: 4.98 ± 1.58 *μ*g/ml) were used as a standard reference in this study. Previous studies revealed that a fruit sample of aqueous extract, water fraction, and ethyl acetate fraction of *Ficus deltoidea* var. *angustifolia* and *Ficus deltoidea* var. *kunstleri* showed a DPPH radical scavenging activity ranges from IC_50_: 111.20–678.18 *μ*g/ml and IC_50_: 148.02–499.93 *μ*g/ml, respectively [61]. The cultivated and wild leaves extracts of *F. deltoidea* have strong DPPH scavenging activity as compared to its stem extract [62]. Abdullah et al. [11] have reported that a good percentage of antioxidant activity of DPPH was found in the methanolic extract of *Ficus deltoidea* var. *trengganuensis*, *Ficus deltoidea* var. *angustifolia*, and *Ficus deltoidea* var. *deltoidea*. Thus, the results of DPPH radical scavenging activity for methanolic *Ficus deltoidea* extracts in this study showed a significant effect, as with previous studies.

The reducing power assay was employed to evaluate the potentiality of antioxidants to donate electrons [63] and the presence of reducers caused an alteration of Fe^3+^/ferricyanide complex to the ferrous form. The color mixture altered from yellow to various shades of green and blue relying on the intensity of reducing the power of each aggregate. The reducing power activity was measured using a Multimode Plate Reader at 700 nm and an increase in absorbance indicated a stronger reductive capability [64]. In this study, the highest reducing power activity was found in FDI (0.24 ± 0.24 mg AAE/g) followed by FDK (0.21 ± 0.02 mg AAE/g), FDB (0.22 ± 0.06 mg AAE/g), FDT (0.11 ± 0.02 mg AAE/g), FDD (0.04 ± 0.02 mg AAE/g), FDM (0.04 ± 0.02 mg AAE/g), and FDA (0.02 ± 0.01 mg AAE/g). The standard curve of ascorbic acid (0.93 ± 0.03 mg AAE/g) was plotted to measure the reducing activity, and quercetin (0.57 ± 0.09 mg AAE/g) was used as the positive control. In previous studies, the reduction capability of *Ficus deltoidea* extract was the highest in FDD followed by FDT and FDA [11], depicting the same trend as in the present findings. However, the results in the present study are in conflict with other studies by Mohd et al. [65] where the reducing power was found to be the highest in FDD followed by FDI, FDK, FDT, FDB, FDA, and FDM.

The phenolic compounds are broadly distributed and are the most ubiquitous groups of secondary plant metabolites that result in beneficial effects on human health. Hakiman and Maziah [66] reported that the antioxidant compounds found in *Ficus deltoidea* leaf extract were known as a nonenzymatic antioxidant (polyphenol, phenolic acid, and flavonoid) and enzymatic antioxidant (ascorbate oxidase, peroxidase, catalase, and ascorbate peroxidase). The phenolic compounds classified as flavonoids (epicatechin, quercetin-3-rutinoside, quercetin 5,4′-di-O-beta-D-glucopyranoside, myricetin, and naringenin) were detected in leaves and figs of aqueous *Ficus deltoidea* var. *kunstleri*, *Ficus deltoidea* var. *angustifolia*, *Ficus deltoidea* var. *deltoidea*, and *Ficus deltoidea* var. *intermedia* extracts [67]. Flavan-3-ol monomer and proanthocyanidins contributed towards 85% of the antioxidant activity found in the aqueous extract of *Ficus deltoidea* [22]. Therefore, phenolic compounds are strongly associated with the effect of antioxidants in the plant sample [66].

The antioxidant capacity of phenolic compounds in plants is primarily due to their redox characteristics, which enable them to serve as reducing agents, singlet oxygen quenchers, hydrogen donators, and metal chelators [68]. The highest phenolic content of *Ficus deltoidea* extracts was found in FDB (299.78 ± 5.85 mg GAE/g) followed by FDI (190.30 ± 3.08 mg GAE/g), FDK (180.47 ± 3.25 mg GAE/g), FDT (163.28 ± 7.83 mg GAE/g), FDD (103.84 ± 5.93 mg GAE/g), FDM (78.44 ± 2.88 mg GAE/g), and FDA (70.90 ± 3.32 mg GAE/g). Quercetin was used as a standard reference and exhibited 1297.94 ± 17.66 mg GAE/g. This study was coherent with the findings by Abdullah et al. [11] where TPC was found to be higher in FDT compared to FDD. A study by Mohd et al. [65] demonstrated the TPC values of the extracts to be in the following order: FDD > FDI > FDK > FDT > FDB > FDM > FDA. This pattern is very similar to the present findings with one exception; in this study, FDB showed the highest activity and FDD was in fifth place. The total phenolic content of methanolic FDA (fruit) was found to be 245.2 mg GAE/g [69]. Misbah et al. [61] reported that for the phenolic content of the fruit, the FDA was higher compared to FDK. The previous results were in contrast with the present findings; it is noted that they were using a different part of the *Ficus deltoidea* plant and with a different extraction solution.

The correlation analysis of TPC, DPPH, reducing power, and antiglycation activity was conducted using Pearson's correlation coefficient. Pearson's correlation coefficient was positively high when 0.6 ≤ *r* ≤ 1 and negatively high when −0.6 ≤ *r* ≤ −1. The results showed that a strong positive correlation between TPC and DPPH was found in FDK (*r* = 1), FDI (*r* = 1), FDA (*r* = 1), and FDD (0.7) where FDK and FDI showed high phenolic content with high DPPH scavenging activity. Meanwhile, the FDA and FDD showed low TPC and antioxidant activity levels. FDT showed a weak correlation at *r* = 0.36 with average levels of TPC and DPPH scavenging activity. There was a negative correlation between total phenolic content and DPPH scavenging effects of FDB and FDM with *r* = −1. Even though FDB had high levels of phenolic content and DPPH activity, FDM had a low level of TPC with low DPPH scavenging activity.

A comparison of TPC to reducing power activity exhibited a strong correlation in FDM (*r* = 1) followed by FDT (*r* = 0.60). Meanwhile, a weak correlation was noted in FDK (*r* = 0.46) and FDB (*r* = 0.36). There was a negative correlation between the assays for FDA (*r* = −0.31), FDI (*r* = −0.66), and FDD (*r* = −0.69), respectively. Researchers [65] have claimed that a high correlation of TPC and reducing power activity was found in *Ficus deltoidea* extracts. In the present study's findings, positive correlations were seen in TPC, DPPH, and reducing power activity, thus indicating a significant contribution of phenolic compounds to both antioxidant assays. It was reported that correlation studies between total phenolic content and free radical scavenging activities of the plant extracts discovered a positive correlation between plant phenolics content and antioxidant activity [61].

A comparison of TPC and antiglycation activity (1.25 mg/mL extract) at 28 days of incubation revealed a positive correlation in some of the extracts. The highest correlation was found in FDM (*r* = 1.0) followed by FDD (*r* = 0.99). Both FDM and FDD showed low phenolic content (compared to other varieties) with moderate antiglycation properties (32.95% and 66.74%, respectively). Even though a weak correlation was seen in FDK (*r* = 0.38) and FDT (*r* = 0.23), they possessed high TPC levels with good inhibition of glycation activity at 65.91% and 64.1% accordingly. Meanwhile, negative correlations were observed in the FDA (*r* = −1.0), FDB (*r* = −1.0), quercetin (*r* = −0.97), and FDI (*r* = −0.37). Apart from that, FDB was observed to have the highest phenolic content and glycation inhibitory activity among the extracts. The antioxidative properties of the phenolic compounds may be a major factor contributing to the prevention of AGE formation in plant extracts [51]. Not only that, a strong correlation was noticed between the polyphenolic content and their ability to inhibit protein glycation [70]. Meanwhile, researchers [71] have reported that polyphenolic compounds in the medicinal plants play a protective role against sugar-induced protein glycation and oxidation. In the present study, *Ficus deltoidea* extracts contained phenolic compounds and possessed a reducing power as well as DPPH radical scavenging activity. *Ficus deltoidea* extracts may reduce the accumulation of advanced glycation end products by scavenging free radicals formed by autooxidation of sugars and/or oxidative degradation of Amadori products.

Our study also demonstrated that the antiglycation and antioxidant activities of *Ficus deltoidea* varieties are in correlation with the total phenolic contents present in the extracts. It was suggested that the synergistic effects of phenolic compounds can be attributed to the antioxidant and antiglycation properties in plant extracts [72]. The antioxidant properties noted in this study indicated that the *Ficus deltoidea* extracts are a significant source of natural antioxidants and their potential could be due to the phenolic contents. Since reactive oxygen species (ROS) are associated with the pathogenesis of inflammatory diseases and diabetes, the free radical inhibitory activity of the plant extracts may be the reason behind their medicinal use in treating a number of disease conditions [73]. Based on its antiglycation and antioxidant (DPPH) activity, FDK was suggested to be the most effective variety compared to others. FDT showed the highest inhibition of AGEs but depicted lower DPPH scavenging activity compared to FDK. Meanwhile, FDB had the highest DPPH scavenging effects but reduced antiglycation activity compared to FDK.

To our knowledge, the study reported here is the most comprehensive comparison of the antioxidant and antiglycation activities among the seven varieties of *Ficus deltoidea* leaf extracts. Therefore, plant extracts with antioxidant and antiglycation properties may offer good remedial potential in reducing the pathogenesis of secondary complications of diabetes.

## 5. Conclusions

The results of this study have revealed that the seven varieties of *Ficus deltoidea* leaf extracts inhibited the formation of AGEs, decreased the levels of fructosamine, increased thiol group levels, and retarded the formation of protein carbonyl. The *Ficus deltoidea* extracts also contained phenolic compounds and possessed antioxidant activities. However, further studies need to be done to identify the active biological constituents of *Ficus deltoidea* that are involved in its activities.

## Figures and Tables

**Table 1:** Total phenolic contents and antioxidant activity of the extracts of *Ficus deltoidea* varieties and quercetin on DPPH scavenging activity, total phenolic content, and reducing power assay.

Sample	DPPH (IC_50_) (*μ*g/ml)	Total phenolic content (mg GAE/g)	Reducing power activity (mg AAE/g)
Quercetin	4.98 ± 1.58	1297.94 ± 17.66	0.57 ± 0.09^*∗∗∗*^
Ascorbic acid	1.3 ± 0.74	—	0.93 ± 0.03
FDT	103.95 ± 9.44	163.28 ± 7.83^*∗∗∗*^	0.11 ± 0.01^*∗∗∗*^
FDK	76.80 ± 4.64	180.47 ± 3.25^*∗∗∗*^	0.21 ± 0.02^*∗∗∗*^
FDI	78.96 ± 6.25	190.30 ± 3.08^*∗∗∗*^	0.24 ± 0.24^*∗∗∗*^
FDD	179.68 ± 6.81	103.84 ± 5.93^*∗∗∗*^	0.04 ± 0.02^*∗∗∗*^
FDA	203.92 ± 0.00	70.90 ± 3.32^*∗∗∗*^	0.02 ± 0.01^*∗∗∗*^
FDB	66.81 ± 4.32	299.78 ± 5.85^*∗∗∗*^	0.22 ± 0.03^*∗∗∗*^
FDM	288.04 ± 11.43	78.44 ± 2.88^*∗∗∗*^	0.04 ± 0.00^*∗∗∗*^

*Note.* The effects of the extracts of *Ficus deltoidea* varieties and quercetin on DPPH scavenging activity, total phenolic content (^*∗∗∗*^*p* < 0.001 compared to quercetin), and reducing power assay (^*∗∗∗*^*p* < 0.001 compared to ascorbic acid). Each value represents the mean ± SD (*n* = 4).

**Table 2:** The correlation study between total phenolic content (TPC) and DPPH assay, total phenolic content (TPC) and reducing power assay, and total phenolic content (TPC) and inhibition of AGE formation of the extracts of *Ficus deltoidea* varieties and quercetin.

Sample	TPC vs DPPH	TPC vs reducing power	TPC vs inhibition of AGE formation
Quercetin	1	−0.1	−0.97
FDT	0.36	0.6	0.23
FDK	1	0.46	0.38
FDI	1	−0.66	−0.37
FDD	0.7	−0.69	0.99
FDA	1	−0.31	−1
FDB	−1	0.36	−1
FDM	−1	1	1

The results were depicted as mean ± SD (*n* = 3).

## Data Availability

The data used to support the findings of this study are available from the corresponding author upon request.
